# Enteroaggregative Shiga toxin-producing *Escherichia coli* of serotype O104:H4 in Belgium and Luxembourg

**DOI:** 10.1002/nmi2.58

**Published:** 2014-08-05

**Authors:** K De Rauw, S Vincken, L Garabedian, E Levtchenko, I Hubloue, J Verhaegen, J Craeghs, Y Glupczynski, J Mossong, D Piérard

**Affiliations:** 1 National Reference Centre for STEC/VTEC, Universitair Ziekenhuis Brussel, Vrije Universiteit Brussel (VUB)Brussels, Belgium; 2 Department of Microbiology, Universitair Ziekenhuis Brussel, Vrije Universiteit Brussel (VUB)Brussels, Belgium; 3 Department of Internal Medicine, Universitair Ziekenhuis Brussel, Vrije Universiteit Brussel (VUB)Brussels, Belgium; 4 Department of Pediatric Nephrology, Universitair Ziekenhuis Leuven, Katholieke Universiteit Leuven (KUL)Leuven, Belgium; 5 Department of Emergency, Universitair Ziekenhuis Brussel, Vrije Universiteit Brussel (VUB)Brussels, Belgium; 6 Department of Microbiology, Universitair Ziekenhuis Leuven, Katholieke Universiteit Leuven (KUL)Leuven, Belgium; 7 Department of Microbiology, Algemeen Ziekenhuis VesaliusTongeren, Belgium; 8 National Reference Centre ESBL and carbapenemase-producing Enterobacteriaceae, CHU Dinant-Godinne|UCL Namur, Université Catholique de Louvain (UCL)Yvoir, Belgium; 9 Surveillance & Epidemiology of Infectious Diseases, National Health LaboratoryDudelange, Luxembourg

**Keywords:** Enteroaggregative *Escherichia coli*, enterohaemorrhagic *E. coli*, gastrointestinal disease, haemolytic uraemic syndrome, Shiga toxin-producing *E. coli*, travel

## Abstract

In 2011, a large outbreak of infections caused by Shiga toxin-producing *Escherichia coli* (STEC) O104:H4 occurred in Germany. This exceptionally virulent strain combined virulence factors of enteroaggregative *E. coli* (EAggEC) and STEC. After the outbreak only a few sporadic cases of infection with this rare serotype were reported, most of which were related to travel to the Middle East or North Africa. Here we describe two cases of enteroaggregative STEC (Agg-STEC) O104:H4 infection that occurred in Belgium in 2012 and 2013 respectively. In both cases travel in a Mediterranean country preceded the infection. The first strain was isolated from the stool of a 42-year-old woman presenting bloody diarrhoea, who had travelled to Tunisia the week before. The second case involves a 14-year-old girl who, upon her return from Turkey to Belgium, suffered from an episode of bloody diarrhoea and haemolytic uraemic syndrome. Extended typing of the isolates with pulsed field gel electrophoresis revealed that the strains were closely related, though not exactly the same as the 2011 outbreak strain. This report supports the previously made hypothesis that Agg-STEC has a human reservoir and might be imported by travellers coming from an area where the pathogen is endemic. Furthermore, it emphasizes the concern that these bacteria may cause future outbreaks as evenly virulent O104:H4 isolates seem to be widespread.

## Introduction

O157:H7 is the most common serotype in Shiga toxin- or Vero(cyto)toxin-producing *Escherichia coli* (STEC/VTEC). A key property of these strains is the production of “Attaching and Effacing” (AE) lesions. STEC of different serotypes can cause severe disease in humans, including haemorrhagic colitis and haemolytic uraemic syndrome (HUS). For this reason, a widely used acronym for these *E. coli* strains is ‘EHEC’ (enterohaemorrhagic *E. coli*). As there is no agreement on the definition of enterohaemorrhagic strains, the acronym ‘AE-STEC’ will be used throughout this paper to refer to Shiga toxin- and AE-lesion-producing *E. coli* strains. AE-STEC O157:H7 have the highest incidence and are the most frequently involved in outbreaks (seropathotype A). AE-STEC of serotypes O26:H11, O103:H2, O111:H-, O121:H19 and O145:H- are also involved in severe human disease and outbreaks, though less frequently (seropathotype B) 1. In 2010, 40.7% of all reported STEC serogroups in Europe were O157. O26 was the most commonly reported non-O157 serogroup (11%) 2.

From May to July 2011, a large outbreak of STEC infections with a serotype that was reported only very rarely before, O104:H4, occurred in Germany. This outbreak differed from previous STEC outbreaks by its sheer size, the large percentage of patients with HUS and the high proportion of adults, mainly women. This exceptionally virulent strain combined virulence factors of enteroaggregative *E. coli* (EAggEC) and STEC making it a mixed enteroaggregative and Shiga toxin-producing *E. coli* (designated here as ‘Agg-STEC’) 1,3. At the same time, a smaller outbreak of Agg-STEC O104:H4 took place in France. Comparison of the back tracing information from the French and German outbreaks led to the conclusion that fenugreek seeds imported from Egypt were the most likely source of infection although the outbreak strain could not be isolated from the seeds 4.

After the 2011 outbreak, only a few sporadic cases of Agg-STEC of serotype O104:H4 were reported. In this paper, we describe two imported cases of Agg-STEC O104:H4 infection that occurred in Belgium after the 2011 outbreak and compare their phenotypic and genetic profiles with the German outbreak strain recovered from a Luxembourg resident who had travelled to the Hamburg area.

## Case Descriptions

The first case is a 42-year-old woman who presented on the first day of September 2012 to the emergency department of the Universitair Ziekenhuis Brussel (Belgium) with complaints of heavy abdominal cramps, nausea and watery diarrhoea. She mentioned the presence of blood in the faeces. She had returned 3 days before from a 1-week journey in Tunisia where diarrhoea started in the morning, just before leaving. She was not aware of other cases. A blood analysis was normal, without signs of HUS (creatinine 0.62 mg/dL, haemoglobin 13.4 g/dL, platelets 393 000/mm³). At physical examination only discrete signs of dehydration were present. She was discharged with dietetic advice and a prescription of Enterol™ (*Saccharomyces boulardii*) and domperidone. She did not consult anymore, as she recovered uneventfully.

The second case involves a 14-year-old girl who was referred from the regional hospital AZ Vesalius to the Universitair Ziekenhuis Leuven (Belgium) in the third week of July 2013 because of abdominal pain, bloody stools, fever and general malaise for 4 days. Her blood examination revealed signs of HUS (anaemia with low haemoglobin 11.3 g/dL, increased levels of lactate dehydrogenase and bilirubin total 10.21 mg/dL and thrombocytopenia with platelet count 27 000/mm³), with presence of schistocytes in peripheral blood smear and decreased kidney function (oliguria, creatinine 1.07 mg/L, urea 68 mg/dL). She was treated supportively with spontaneous recovery of her kidney function. No dialysis was needed. The patient had travelled to Turkey during the first 2 weeks of July where she developed watery diarrhoea that spontaneously resolved after 5 days. Upon her return to Belgium she suffered from a new episode of diarrhoea, this time with the presence of blood in stools, and she developed HUS 6 days later.

The strains isolated from these patients were compared with an isolate related to the German outbreak. A 51-year-old woman residing in Luxembourg travelled for several days during the second week of May 2011 in the Hamburg region in Germany. Hamburg was the German state with the highest incidence (10 cases per 100 000 population) of reported HUS during the outbreak 3. A few days after her return, she developed diarrhoea, which gradually worsened until she noticed blood in her faeces and consulted a physician who prescribed a stool culture. The patient recovered without developing HUS.

## Methods and Results

In both Belgian cases a STEC O104:H4, referred to as EH2211 and EH2303, respectively, was recovered out of the stools by using methods described elsewhere 5. Slide agglutination was performed with the K9 antiserum (Statens Serum Institut, Copenhagen, Denmark), indicative of the O104 serogroup 6. The full serotype was confirmed by a positive PCR result for O104 (*wzxO104*) and H4 (*fliCH4*) 7. Both strains grew on sorbitol–MacConkey agar with cefixime and tellurite (SMAC-CT), fermented sorbitol and were β-glucuronidase positive. By contrast with EH2211, EH2303 did not ferment lactose. A PCR was positive for *aaiC* and *aggR* genes 8, but negative for the STEC virulence genes intimin (*eaeA*) and enterohaemolysin (*hlyA*) 9. Both strains possessed the Shiga toxin 2a (*stx2a*) subtype, as described by Scheutz *et al*. 10. Disc diffusion susceptibility testing according to EUCAST recommendations (or CLSI when no breakpoints were available from the former) was performed using antibiotics selected by the Programme for Food- and Waterborne Diseases and Zoonoses (FWD) and revealed that strain EH2211 had an intermediate susceptibility to nalidixic acid and was resistant to ampicillin and trimethoprim. EH2303 on the other hand was resistant to ampicillin, amoxicillin/clavulanic acid, cefazolin, cefuroxime, cefotaxime, ceftriaxone, ceftazidime, cefepime, aztreonam, sulfonamides, trimethoprim and nalidixic acid. No extended-spectrum β-lactamase (ESBL) was detected for EH2211 according to CLSI screening and confirmatory tests; but EH2303 tested ESBL positive, further characterized by the National Reference Centre for multiresistant *Enterobacteriaceae* as CTX-M-15; a TEM-1 β-lactamase was also present.

Pulsed-field gel electrophoresis using *Xba*I was performed using the PulseNet standard operating procedure 11 to compare these two Agg-STEC O104:H4 strains with C110638, an Agg-STEC O104:H4 from Luxembourg linked to the 2011 German outbreak, and EH1651, an unrelated STEC O104:H2 strain included in the study of Miko *et al*. 12 (Table1).

**Table 1:** Features of the strains analysed in this publication

Strain	Country of isolation	Year of isolation	Travel related	Serotype	Stx-subtype	Virulence genes				ESBL	Lactose
						AE-STEC		EAggEC			
						*eaeA*	*hlyA*	*aaiC*	*aggR*		
EH1651	Belgium	2008	Morocco	O104:H2	Stx2b	−	−	−	−	−	+
C110638	Luxembourg	2011	Hamburg, Germany	O104:H4	Stx2a	−	−	+	+	+	+
EH2211	Belgium	2012	Tunisia	O104:H4	Stx2a	−	−	+	+	−	+
EH2303	Belgium	2013	Turkey	O104:H4	Stx2a	−	−	+	+	+	−

EH1651 is a historical O104 Shiga toxin-producing *Escherichia coli* (STEC) strain from our collection and was included in this study as a control; AE-STEC, ‘Attaching and Effacing’-lesions- and Shiga toxin-producing *E. coli*; EAggEC, enteroaggregative *E. coli*; ESBL, extended-spectrum β-lactamase; Stx, Shiga toxin.

**Figure 1:**
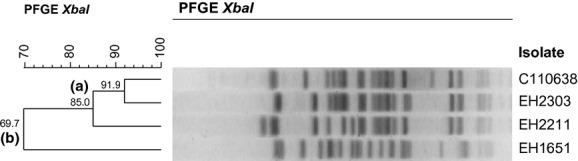
Pulsed-field gel electrophoresis *Xba*I profiles of EH1651 (O104:H2), EH2211 (O104:H4), EH2303 (O104:H4) and C110638 (O104:H4). The dendrogram was generated with the BioNumerics 6.5 software (Applied Maths, Sint-Martens-Latem, Belgium) using Dice coefficients and the unweighted pair group method with arithmetic mean. Using a cut-off value of 80% similarity two clusters, A and B, can be detected.

Strain EH2303 clustered closest to the 2011 outbreak strain with 91.9% similarity. Using a cut-off value of 80% similarity the three O104:H4 strains could be divided in the same cluster A, whereas strain EH1651 showed only 69.7% similarity and formed a separate group B (Fig.1).

## Discussion

Although O104:H4 is a rare serotype in humans, a few sporadic infections caused by *E. coli* O104:H4 took place before 2011. STEC O104:H4 caused a case of HUS in South Korea (2004), but this strain was not enteroaggregative 13. EAggEC O104:H4 has been associated with human diarrhoea in South Africa (2007, 2009 and 2010) and Canada (2010) 14,15. Agg-STEC O104:H4 have been isolated from patients with HUS in Germany (2001), France (2009) and Italy (2009); from two patients with bloody diarrhoea in the Republic of Georgia (2009); from a patient with haemorrhagic colitis in France (2004); and from patients in Norway (2006) and Sweden (2010) 16–20. After the German and French outbreaks, cases of Agg-STEC occurred in France, Germany and Denmark 21,22. HUS caused by Agg-STEC other than O104:H4 has been reported in France (1992), Japan (1999) and Ireland (2012). The strains belonged to serotypes O111:H2, O86:HNM and O111:H21, respectively 23–25 (Table2).

**Table 2:** Overview of enteroaggregative Shiga toxin-producing *Escherichia coli* infections described in literature

References	Year of isolation	Country of isolation	Serotype	Source of infection	Sporadic or outbreak related	Imported?	Disease	Stx	ESBL	Lactose
23,30	1992	France	O111:H2	Probably person to person transmission; no food or drinks found	Outbreak (*n* = ?; 8 strains with Agg phenotype)	NR	D, BD, HUS	Stx2	NR	NR
24	1999	Japan	O86:HNM	NR	Sporadic	NR	HUS, encephalopathy	Stx2	NR	NR
16,31	2001	Germany	O104:H4	NR	Sporadic	NR	HC, HUS	Stx2	NR	NR
18	2004	France	O104:H4	NR	Sporadic	NR	HC	Stx2	−	NR
16	2006	Norway	O104:H4	NR	Sporadic	NR	D	Stx2	NR	NR
18	2009	France	O104:H4	NR	Sporadic	NR	HUS	Stx2	−	NR
19	2009	Italy	O104:H4	Probably person to person transmission	Sporadic	Tunisia, travel	BD, HUS	Stx2	−	NR
6,32	2009	Republic of Georgia	O104:H4	NR	Outbreak (*n* = ?; 2 strains with Agg phenotype)	NR	BD	Stx2a	−	+
20	2010	Sweden	O104:H4	NR	Sporadic	Tunisia, travel	NR	Stx2	−	NR
3,6	2011	Germany	O104:H4	Probably seeds, grown to fenugreek sprouts	Outbreak (*n* = 3816)	Probably Egyptian seeds	D, BD, HUS	Stx2	+	+
3,33	2011	France	O104:H4	Probably seeds, grown to fenugreek sprouts	Outbreak (*n* = 24)	Probably Egyptian seeds	D, BD, HUS	Stx2	+	+
22	2011	France	O104:H4	NR	Sporadic	NR	HUS	Stx2	−	NR
22	2011	France	O104:H4	NR	Sporadic	NR	HUS	Stx2	−	NR
22	2011	France	O104:H4	NR	Sporadic	Turkey, travel	HUS	Stx2	−	NR
22	2011	France	O104:H4	NR	Sporadic	NR	HUS	Stx2	−	NR
22	2011	France	O104:H4	NR	Sporadic	North Africa, travel	HUS	Stx2	−	NR
21	2011	Germany	O104:H4	NR	Sporadic	Turkey, travel	BD	Stx2	−	NR
21	2011	Germany	O104:H4	NR	Sporadic	Turkey, travel	BD	Stx2	−	NR
21	2011	Denmark	O104:H4	NR	Sporadic	Turkey, travel	D	Stx2	−	NR
21	2011	France	O104:H4	NR	Outbreak (*n* = 8)	Turkey, travel	D, BD, HUS	Stx2	−	NR
25	2012	Ireland	O111:H21	NR	Outbreak, household (*n* = 3)	−	D, HUS	Stx2c	−	NR
This report	2012	Belgium	O104:H4	NR	Sporadic	Tunisia, travel	BD	Stx2a	−	+
This report	2013	Belgium	O104:H4	NR	Sporadic	Turkey, travel	HUS	Stx2a	+	−

NR, not reported; −, negative; +, positive; D, diarrhoea; BD, bloody diarrhoea; HC, haemorrhagic colitis; HUS, haemolytic uraemic syndrome; ESBL, extended-spectrum β-lactamase; Stx, Shiga toxin.

Common characteristics of EH2211, EH2303 and the German outbreak strain include: ability to grow on SMAC-CT agar; positive PCR results for *aaiC*, *aggR*, *stx2a*, *wzxO104* and *fliCH4;* negative PCR results for *eaeA*, *hlyA* and s*tx1*; agglutination with K9 antiserum; fermentation of sorbitol and production of β-glucuronidase. The outbreak strain and strain EH2303 were both multiresistant and produced an ESBL CTX-M-15 while EH2211 was susceptible to most tested antibiotics 6,22,26. A remarkable characteristic of strain EH2303 is the fact that it did not ferment lactose. To the best of our knowledge, this is the first lactose-negative Agg-STEC O104:H4 strain ever reported.

Strain EH2303 shows more similarity to the strain from Luxembourg than EH2211 based on their pulsed field gel electrophoresis profiles. Closely related, but not identical, pulsed field gel electrophoresis *Xba*I patterns of *E. coli* O104:H4 strains isolated before and after the German 2011 outbreak have already been described 12,21.

In the cases described in this paper, travel to Turkey and Tunisia preceded the infection with Agg-STEC O104:H4, suggesting that the infection was acquired in these countries. Indeed, other sporadic cases of infection with *E. coli* O104:H4, as well as an outbreak associated with travel to Turkey or North Africa, have been reported in Europe. According to the information reported to European Centre for Disease Prevention and Control, four of the STEC O104 cases in the EU Member States in the period 2004–2010 were travel-related; the countries of origin of infection were Afghanistan, Egypt, Tunisia and Turkey. One of those was of the serotype O104:H4; a Finnish case with infection acquired in Egypt 26. Infections of Agg-STEC O104 were noted in Italy (2009) and Sweden (2010). As in the present case, they were linked to travel to Tunisia 19,20. In September 2011, an outbreak of HUS due to Agg-STEC O104:H4 occurred among French tourists returning from Turkey. Around that same period, two patients from Germany and one from Denmark were diagnosed with an Agg-STEC O104:H4 infection, also after travel to Turkey 21. Out of four sporadic isolates of *E. coli* O104:H4 from children with HUS in France, one was associated with recent travel to North Africa 22.

Whereas AE-STEC is clearly a zoonosis, mainly related to ruminants, no animal reservoir has been reported for EAggEC nor Agg-STEC. Studies conducted in cattle in Germany and France during the 2011 outbreak were not able to detect the presence of the outbreak strain, indicating that cattle are not the reservoir of Agg-STEC 27,28. As no other natural reservoir has been established, it has been hypothesized Agg-STEC has a human reservoir and might be imported into the European Union by travellers or migrants coming from an area where the pathogen is endemic 29. Without an animal reservoir, it is not expected that Agg-STEC strains can be maintained in countries with high standards of hygiene.

Comparative genomics of *E. coli* O104:H4 strains isolated before, during and after the 2011 outbreak provided us with better insight into the evolution of this serotype. Genomic analysis of a number of post-outbreak O104:H4 strains has shown that they are not derived from the 2011 outbreak but share a recent common ancestor 22. A nearly identical Shiga toxin-carrying prophage was found in several of the historical O104:H4 strains, which suggests that the prophage was transferred into the O104:H4 population at least a decade ago. On the other hand, acquisition of the antibiotic resistance plasmid pESBL and gene cassettes for antibiotic resistances seems to be one of the most recent events in the evolutionary history 20,22. The two cases reported here had differing antibiotic resistance profiles. Strain EH2211 was susceptible to most antibiotics, whereas EH2303 was multiresistant, as was the 2011 outbreak strain, and carried a CTX-M-15 type ESBL.

In conclusion, the cases described here support the previously expressed concern that these bacteria may cause future severe outbreaks as evenly virulent, though not exactly the same, O104:H4 isolates seem to be widespread.
